# Description and Demonstration of the Coupled Community Earth System Model v2 – Community Ice Sheet Model v2 (CESM2‐CISM2)

**DOI:** 10.1029/2020MS002356

**Published:** 2021-06-24

**Authors:** Laura Muntjewerf, William J. Sacks, Marcus Lofverstrom, Jeremy Fyke, William H. Lipscomb, Carolina Ernani da Silva, Miren Vizcaino, Katherine Thayer‐Calder, Jan T. M. Lenaerts, Raymond Sellevold

**Affiliations:** ^1^ Department of Geoscience and Remote Sensing Delft University of Technology Delft The Netherlands; ^2^ Climate and Global Dynamics Laboratory National Center for Atmospheric Research Boulder CO USA; ^3^ Department of Geosciences University of Arizona Tucson AZ USA; ^4^ Associated Engineering Group Ltd Calgary AB Canada; ^5^ Department of Atmospheric and Oceanic Sciences University of Colorado Boulder Boulder CO USA

**Keywords:** ice sheet modeling, Earth system modeling, model coupling

## Abstract

Earth system/ice‐sheet coupling is an area of recent, major Earth System Model (ESM) development. This work occurs at the intersection of glaciology and climate science and is motivated by a need for robust projections of sea‐level rise. The Community Ice Sheet Model version 2 (CISM2) is the newest component model of the Community Earth System Model version 2 (CESM2). This study describes the coupling and novel capabilities of the model, including: (1) an advanced energy‐balance‐based surface mass balance calculation in the land component with downscaling via elevation classes; (2) a closed freshwater budget from ice sheet to the ocean from surface runoff, basal melting, and ice discharge; (3) dynamic land surface types; and (4) dynamic atmospheric topography. The Earth system/ice‐sheet coupling is demonstrated in a simulation with an evolving Greenland Ice Sheet (GrIS) under an idealized high CO_2_ scenario. The model simulates a large expansion of ablation areas (where surface ablation exceeds snow accumulation) and a large increase in surface runoff. This results in an elevated freshwater flux to the ocean, as well as thinning of the ice sheet and area retreat. These GrIS changes result in reduced Greenland surface albedo, changes in the sign and magnitude of sensible and latent heat fluxes, and modified surface roughness and overall ice sheet topography. Representation of these couplings between climate and ice sheets is key for the simulation of ice and climate interactions.

## Key Points

- We present a bi‐directionally coupled ice sheet and Earth system model including advanced representation of surface snow/ice processes
- The surface mass balance is calculated in the land component with an energy balance scheme and downscaled to the ice‐sheet component via elevation classes
- Ice sheet meltwater and solid discharge are passed to the ocean component; changes in ice sheet area and topography to land and atmosphere

## 1 Introduction

Land ice exists in the Earth system where perennial snow fields can form and develop into flowing ice masses (Agassiz, 1840). Once land ice has formed, the climate modulates ice dynamics by controlling processes of mass loss and gain at the ice sheet boundaries (Nye, 1963). Subsequently, as ice sheets evolve, they interact with the climate in ways that modify their own evolution (Fyke et al., 2018). The Earth system contains many such feedback mechanisms, including both positive (amplifying) and negative (moderating) processes. Feedback mechanisms between ice sheets and climate have been extensively studied. These include feedbacks between melt and albedo (Box et al., 2012), elevation and surface mass balance (SMB, the difference between accumulation and ablation at the surface) (Edwards et al., 2014), SMB and ice discharge (Goelzer et al., 2013; Lipscomb et al., 2013), ice‐sheet runoff and calving, ocean circulation, sea ice, ice‐sheet climate (Day et al., 2013), and ice‐sheet topography and atmospheric circulation (Ridley et al., 2005). Despite this body of work, much remains to be understood in the field of ice‐sheet/Earth system feedbacks (Fyke et al., 2018)—including the discovery of new feedback loops. Lack of understanding stems in part from the lack of fully coupled ice‐sheet/Earth system models.

The modeling of ice‐sheet response to climate change, and the response of the Earth System to ice sheet change, is an active, challenging area of research (Vizcaino, 2014). This research is largely motivated by the urgent need to better constrain projections of anthropogenic sea‐level rise. For example, the Greenland Ice Sheet (GrIS) and Antarctic Ice Sheet (AIS) have been losing mass at an accelerating rate in the last several decades (Shepherd et al., 2019; Tapley et al., 2019; Velicogna et al., 2014, 2020), and are now major contributors to global mean sea‐level rise. Ice‐sheet/Earth system interactions are a key aspect of this accelerating trend and must be studied in the coupled context over decadal to centennial timescales to develop robust future sea‐level projections in support of climate understanding, mitigation, and adaptation.

Previous work on integrating ice sheets into Earth System Models (ESMs) for present‐day ice sheet configurations has been done byfor example, Lipscomb et al. (2013), Lofverstrom et al. (2020), Mikolajewicz et al. (2007), Ridley et al. (2005), and Vizcaino et al. (2008, 2014). Work on paleo climates, which usually spans longer time scales, has been done with ESMs of Intermediate Complexity (EMICs; Fyke et al., 2011; Liakka et al., 2012), and ESMs using asynchronous climate/ice‐sheet coupling (Gregory et al., 2012; Lofverstrom & Liakka, 2018; Lofverstrom et al., 2015).

Here, we present the coupling between the Community Earth System Model version 2 (CESM2) and the Community Ice Sheet Model version 2 (CISM2). This work extends preliminary coupling efforts with CESM1 and the Glimmer‐CISM ice sheet model (Rutt et al., 2009), which focused on the development of the SMB calculation and application of this (one‐way) coupled model simulation the GrIS response to future climate change (Lipscomb et al., 2013). CESM2‐CISM2 is designed to interactively simulate the effects of climate on largely terrestrial ice sheets (and vice versa). At present, the main application of the model is to project future multi‐century‐scale mass loss of the GrIS in the broader Earth system and its contribution to global mean sea‐level change.

Within the framework of the Ice Sheet Model Intercomparison Project for CMIP6 (ISMIP6) (Eyring et al., 2016; Nowicki et al., 2016), this study and a companion paper on model spin‐up (Lofverstrom et al., 2020) serve as the primary background documentation for coupled “AOGCM‐ISM” (atmosphere‐ocean general circulation model—ice sheet model) ISMIP6 experiments using CESM2 (Muntjewerf, Petrini, et al., 2020; Muntjewerf, Sellevold, et al., 2020). Beyond ISMIP6, CESM2‐CISM2 is intended to simulate past, present, and future climates, in which ice sheet dynamics and ice‐sheet/Earth system interactions are considered important aspects of the overall system.

Interactive coupling of the AIS is not supported in the model versions that are described here and used for coupled ISMIP6 simulations. The main challenges involve ocean interactions with marine‐based ice, which are essential for modeling the AIS. Work is under way to support interactive coupling with the AIS and paleo ice sheets, in addition to the GrIS, in future versions of CESM.

This study is organized as follows. Section 2 describes the model and its components. Section 3 describes the coupling between CESM2 and CISM2. Section 4 demonstrates the coupled CESM2‐CISM2 for the GrIS with a pre‐industrial simulation and a simulation under an idealized scenario of high atmospheric CO_2_. Section 5 discusses the novelty of the coupled model and the potential for future developments. Finally, Section 6 presents primary conclusions.

## 2 Model Description

CESM2 is a state‐of‐the‐art, coupled ESM primarily maintained at the National Center for Atmospheric Research (NCAR). With atmosphere, land, ocean, sea‐ice, and ice sheet components that interactively exchange state information at runtime, CESM2 allows for realistic simulations of Earth's climate (Danabasoglu et al., 2020). The sections below provide detailed descriptions of the physics and interactions that are relevant for realistic Earth system/ice‐sheet coupling. A comprehensive description of CESM2 can be found in Danabasoglu et al. (2020).

### 2.1 The Atmosphere Model

Atmospheric processes are simulated by the Community Atmosphere Model 6 (CAM6), using a finite‐volume dynamical core (Gettelman et al., 2019; Lin & Rood, 1997). The model operates on a nominal 1° (0.90° latitude × 1.25° longitude) horizontal grid and 32 levels in the vertical with the upper boundary at 3.6 hPa (∼40 km). CAM6 includes substantial improvements of every atmospheric physics parameterization compared to its predecessor CAM5, except for radiative transfer. The Cloud Layers Unified by Binormals (CLUBB; Bogenschutz et al., 2013) scheme has replaced earlier schemes for boundary layer turbulence, shallow convection, and cloud macrophysics. The two‐moment prognostic cloud microphysics scheme (MG2; Gettelman et al., 2015) has been updated. The major innovation in MG2 is to carry prognostic precipitation species—rain and snow—in addition to cloud condensates. MG2 interacts with the MAM4 aerosol microphysics scheme (Liu et al., 2015) to calculate condensate mass fractions and number concentrations. The improved cloud microphysics reduce cloud biases over Greenland, including the representation of cloud liquid water and longwave cloud forcing (Lenaerts et al., 2020). Also, parameterizations of subgrid‐scale surface drag have been modified from CAM5. First, topographic orientation (ridges) and low‐level flow blocking effects have been incorporated into the orographic gravity wave scheme (Scinocca & McFarlane, 2000). Second, the boundary layer form drag is now parameterized with the scheme of Beljaars et al. (2004), which improves the representation of orographic precipitation (notably over southeast Greenland), near‐surface wind, and turbulent heat and moisture fluxes (van Kampenhout et al., 2020).

### 2.2 The Ocean Model

Ocean processes are simulated by the Parallel Ocean Program Version 2 (POP2; Danabasoglu et al., 2012; Smith et al., 2010). POP2 runs on a nominal 1° horizontal grid with a displaced pole over Greenland to avoid grid singularities in the Arctic Ocean. This has the additional benefit of relatively high effective resolution around Greenland and in the deep water formation regions in the Labrador Seas. The vertical *z*‐coordinate is discretized in 60 levels, with uniform 10 m spacing in the upper 160 m, increasing to 250 m in the deep ocean. The ocean model conserves volume.

### 2.3 The Land Model

Land processes are simulated by the Community Land Model Version 5 (CLM5; Lawrence et al., 2019), including a wide range of biogeophysical and biogeochemical processes and snow hydrology. CLM5 uses the same nominal 1° horizontal grid as CAM6. To represent spatial heterogeneity in the landscape, CLM5 uses a fractional approach where calculations are carried out over a hierarchy of surface types. The uppermost level of the hierarchy is the *land unit*; each grid cell is divided into fractions representative of glacier, lake, wetland, urban, vegetated, and crop surfaces. The second, *column* level captures heterogeneity in state variables within each land unit. In the case of the glacier land unit, columns are defined based on surface elevation. In the following, these columns are referred to as elevation classes (ECs; see Lipscomb et al., 2013). Finally, vegetated land units have *Plant Functional Types (PFTs)* that allow for a variety of crop and vegetation types, including bare ground.

Vertical discretization is different between soil and snow within the model: the soil column has a fixed number of layers, while snow/firn has a variable number of layers and a maximum allowed snow depth H_*max*_. Vegetated land units have 15 layers, the lower 5 being specified as bedrock. The subsurface of glacier land units consists of 15 ice layers that are fully frozen and impervious to water infiltration. Snow/firn is represented by up to 12 snow layers, depending on the total thickness. Each snow layer has a prescribed maximum thickness that is larger for deeper layers. When a snow layer exceeds its maximum thickness, excess mass is transferred downward. If there is no layer beneath, a new layer is initialized. The CLM5 default H_*max*_ is 10 m water equivalent (mWE). In areas with perennial snow cover, this depth allows the development of a firn layer that is sufficiently deep for firn hydrological processes, such as refreezing and storage of surface melt water. From fresh snow accumulation, the model simulates compaction of snow into firn, with prognostic density and “pore” space, considering the effects of snow mass overburden, snow metamorphism, and enhanced wind‐driven surface compaction (Van Kampenhout et al., 2017).

Snow albedo is calculated using the SNow, ICe, and Aerosol Radiation (SNICAR) model, which accounts for vertically resolved solar absorption, snow grain size evolution, and snow with impurities from aerosols (Flanner & Zender, 2006; Flanner et al., 2007). SNICAR simulates the spectral albedo of snow in a multilayer approximation, to account for vertically heterogeneous properties and heating. Albedo is computed for five spectral bands, accounting for grain size changes and aerosol heating and scattering. The resulting albedo is averaged to the visible and near‐infrared bands used in CLM. The capability to simulate aerosol deposition on snow allows for studies of its impact on surface albedo and snow melt (e.g., Li & Flanner, 2018).

Heat and water fluxes and phase changes between liquid and solid water are calculated in all vertical layers for each column and/or PFT given the overlying atmospheric heat fluxes as upper boundary condition. The lower boundary is assumed to have a net‐zero heat flux. CLM5 calculates the surface energy fluxes separately for the snow‐covered and snow‐free fractions of a land unit.

Surface runoff is routed to the ocean by the Model for Scale Adaptive River Transport (MOSART, Li et al., 2015). Transport is based on present‐day topography gradients, and is simulated with time‐varying velocities and channel water depths.

### 2.4 The Sea‐Ice Model

Sea ice is represented in CESM by the Los Alamos National Laboratory sea ice model, version 5 (CICE5; Hunke et al., 2017). Sea‐ice dynamics are determined by horizontal transport, ridging, and elastic‐viscous‐plastic rheology. The sea‐ice temperature and salinity profiles are resolved in eight layers in the vertical for each thickness category in an ice thickness distribution. Also, a three‐layer snow model calculates the vertical temperature distribution in the snow pack on the sea ice. The horizontal grid is shared with the ocean component.

### 2.5 The Land‐Ice Model

Ice sheets are simulated by the CISM2 (Lipscomb et al., 2019). CISM2 is a parallel, three‐dimensional thermomechanical ice sheet model that can solve several approximations of the Stokes equations for incompressible viscous flow. The default ice‐flow solver for Greenland simulations in CESM2 is a depth‐integrated higher‐order approximation based on Goldberg (2011). By using a depth‐integrated effective viscosity, the model achieves accuracy close to that of the higher‐order Blatter‐Pattyn approximation (Blatter, 1995; Pattyn, 2003), but at much lower computational cost. At present, the Greenland domain is a 4‐km rectangular grid based on a polar stereographic projection, using 11 terrain‐following sigma levels in the vertical. Basal sliding is parameterized in CESM with a pseudo‐plastic sliding law as described by Aschwanden et al. (2016). In standard Greenland simulations, ice discharge at marine margins uses a flotation criterion, where all floating ice immediately calves to the ocean. CISM2.1 also supports more advanced calving schemes, potentially more appropriate for ice sheets with large ice shelves.

## 3 Earth System/Ice‐Sheet Coupling

**Figure 1:**
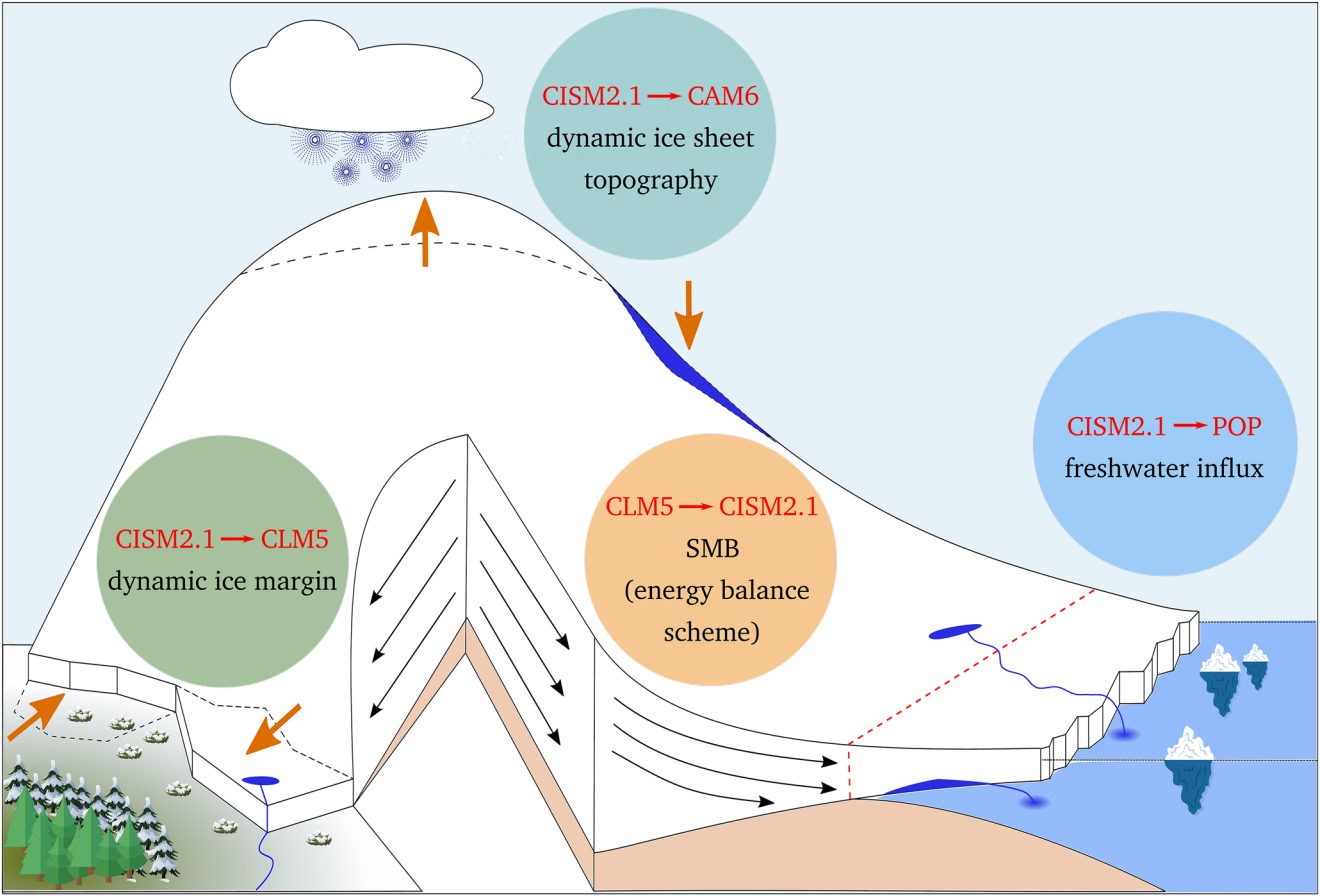
Schematic of four elements of coupling between ice sheets and other Earth system components, courtesy of M. Petrini.

The description of the coupling between CESM2 and CISM2 is organized in four sub‐sections (Figure 1). Support for interactive, time‐evolving ice sheets is currently limited to Greenland. Glaciated regions elsewhere (i.e., the AIS and smaller ice caps and mountain glaciers) are treated as in the default CESM2 configuration with prescribed ice sheets. See for more details the CESM2 Land Ice technical documentation (Leguy et al., 2018) and the CLM5 technical documentation (Lawrence et al., 2020).

### 3.1 Surface Mass Balance Calculation

The SMB calculation is done in CLM5 on multiple elevation classes, with melt calculated from the surface energy balance. The SMB is defined as the difference between accumulation from precipitation, and surface loss from runoff and sublimation:

(1) SMB=Precipitation−Runoff−Sublimation,

Precipitation can fall as either snow or rain. While snowfall contributes directly and positively to the SMB, rainfall can only contribute positively if it refreezes within the snow pack. Otherwise, it contributes to runoff and is routed together with (non‐refrozen) melt water to the ocean by the river model (MOSART). The SMB can therefore be reformulated as the sum of snowfall and refrozen rainfall, minus the sum of melt and sublimation:

(2) SMB=Snowfall+Refreezing−Sublimation−Melt.

Drifting snow processes (Lenaerts et al., 2012) are not yet accounted for in CESM2. Melt energy is calculated from the sum of net surface radiation, latent and sensible turbulent surface fluxes, and ground heat fluxes at the atmosphere‐snow interface (Lawrence et al., 2019):

(3) $$E_{M}=SW_{net}+LW_{net}+LH+SH+GH$$

where *E*_*M*_ denotes available melt energy, *SW*_*net*_ is the net shortwave radiation at the surface, *LW*_*net*_ is the net longwave radiation, *LH* is the latent heat flux, *SH* is the sensible heat flux, and *GH* is the ground heat flux, all in W m^−2^.

The surface energy and mass budgets of ice sheets depend strongly on elevation (Hermann et al., 2018; Van de Wal et al., 2012). However, differences in horizontal resolution between CISM2 (4 km) and CLM5 (nominal 1°) imply that multiple CISM2 grid cells exist within each CLM5 cell. This discrepancy in resolution is particularly challenging around the steep ice sheet margins, where the coarse CLM5 resolution makes it difficult to resolve gradients in the SMB. To overcome this challenge, CLM5 uses multiple elevation classes (ECs; Fyke et al., 2011; Lipscomb et al., 2013; Sellevold et al., 2019; Vizcaino et al., 2014) to account for subgrid‐scale variations in elevation over glaciated land units. This method bins the glaciated land unit fraction of each CLM5 grid cell based on the resolved topography in the higher‐resolution ice‐sheet model. For each EC, surface energy fluxes and their impact on SMB are calculated independently by downscaling atmospheric variables at the CLM5‐CAM6 coupling frequency (every half hour in the current model configuration). The CLM5 grid cell temperature is downscaled to the EC elevation using a uniform lapse rate (by default −6 K km^−1^). Model sensitivity to the lapse rate choice was assessed in Sellevold et al. (2019). With the fixed lapse rate and the assumption of vertically uniform relative humidity, EC‐specific potential temperature, specific humidity, air density, and surface pressure are determined. To tune the magnitude of the elevation feedback, the incoming longwave radiation can optionally be downscaled using a default lapse rate of −32 W m^−2^ km^−1^ (Van Tricht et al., 2016). Finally, precipitation is partitioned into rain or snow according to the elevation‐corrected near‐surface temperature. Precipitation is assumed to be 100% snow (frozen) if the downscaled surface temperature is below −2°C, and rain (liquid) if the temperature is above 0°C. Between these temperatures, precipitation falls as a mix of rain and snow based on a linear ramping relationship. To conserve energy, a sensible heat flux is applied at the surface to compensate for the latent energy absorbed or released in rain‐snow conversion.

**Figure 2:**
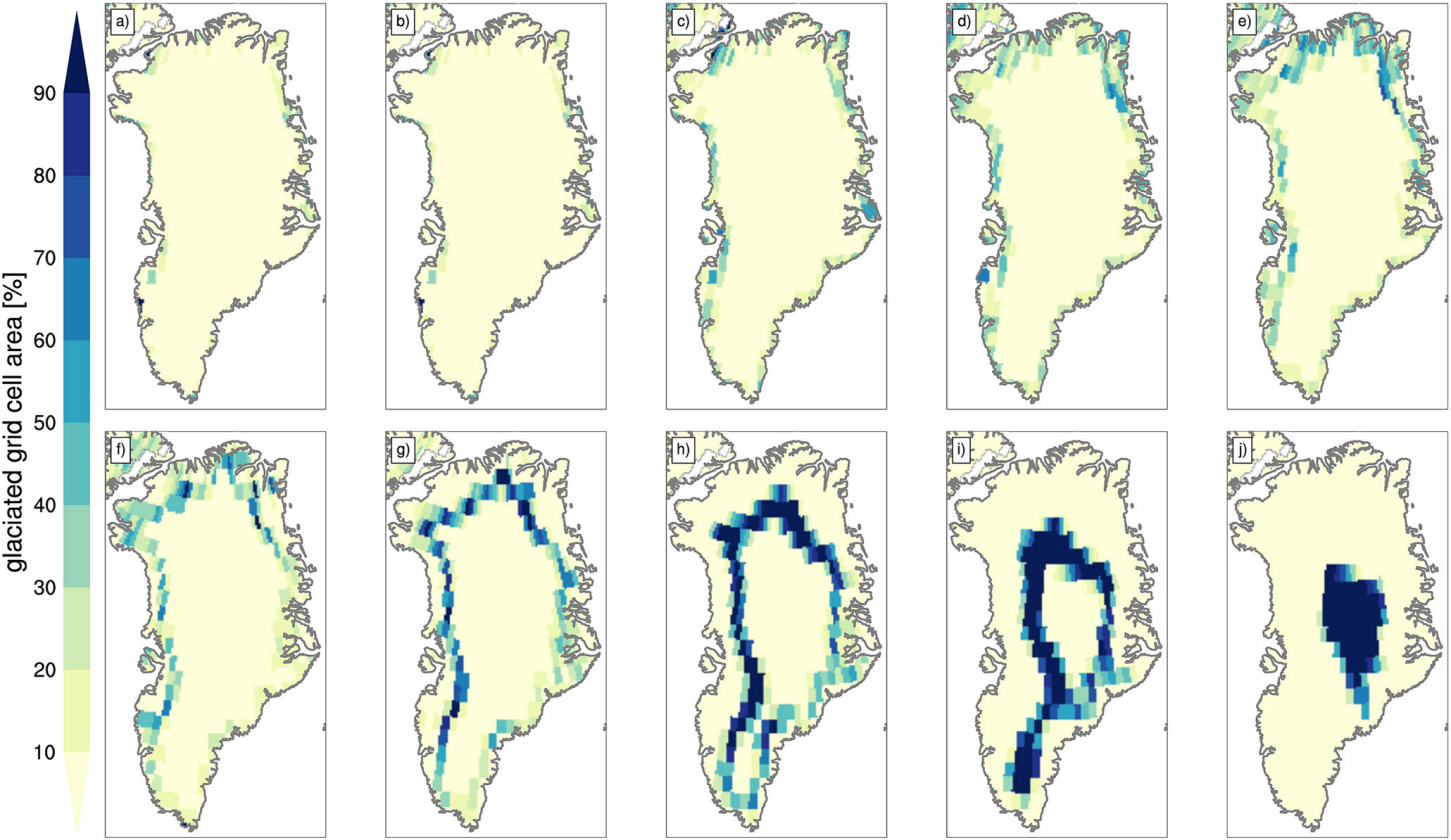
Area percentage of glacier elevation classes in the glaciated fraction of CLM5 land‐model grid cells, for the default 10 elevation bins: (a) EC1: 0–200 m; (b) EC2: 200–400 m; (c) EC3: 400–700 m; (d) EC4: 700–1,000 m; (e) EC5: 1,000–1300 m; (f) EC6: 1,300–1,600 m; (g) EC7: 1,600–2,000 m; (h) EC8: 2,000–2,500 m; (i) EC9: 2,500–3,000 m; (j) EC10: 3,000–10,000 m. The ice sheet topography is from the pre‐industrial simulation described in Section 4. CLM5, Community Land Model Version 5.

Elevation classes allow to account for the impact of subgrid‐scale variations in topography on the SMB and surface energy balance calculations. The model uses 10 elevation classes, with boundaries at 0, 200, 400, 700, 1,000, 1,300, 1,600, 2,000, 2,500, 3,000, and 10,000 m. An additional vegetated EC allows snow to accumulate and form glacier ice in previously non‐glaciated grid cells. Within each elevation class, the “representative” (or actual) elevation is set as the mean of all CISM grid cell surface elevations falling within it. Typically, one or two elevation classes are sufficient to represent the distribution of ice sheet elevations within a CLM5 grid cell in the relatively flat regions of the ice sheet interior. Conversely, more elevation classes are needed to capture topographic variation on the steep ice sheet margins. The remaining ECs in a grid cell are virtual; that is, their area fraction is zero. SMB calculations in virtual ECs allow to realistically initialize a temperature profile when an EC first acquires a nonzero area value. The elevation classes calculations are done in a predefined domain. Figure 2 shows how much area each EC covers on the CLM grid for a pre‐industrial, equilibrated GrIS (Lofverstrom et al., 2020) (see Section 4).

Physically, the interface between snow and ice is characterized as the point at which densities of solid ice are reached, and no additional snow compaction as a result of overburden pressure can occur. In CLM5, the conversion of snow to ice is based on depth; any snow accumulation exceeding a prescribed threshold (10 mWE by default) is assumed to turn to ice. In CESM simulations without interactive ice sheets, this ice is sent to the ocean as a solid runoff flux, but in coupled CESM‐CISM runs, it is sent to the ice sheet model as accumulation (i.e., a positive SMB). An equal amount of mass is then removed from the lowest snow layer in CLM5 to allow refreshing of surface snow density and albedo. Mass variations in snow and firn layers are only considered in the land model, while variations in ice mass “belong” to the ice sheet model. Therefore, the SMB sent from CLM5 to CISM2 is defined as the difference between (annually integrated) ice accumulation and ablation. Accumulation is the sum of snowfall and refrozen rain wherever the maximum allowed CLM5 snow depth is reached (10 mWE by default). An equal amount of mass is then removed from the lowest snow layer to allow refreshing of surface density and albedo. The annual integrated melt that takes place on bare ice (which may be exposed after the snow pack has disappeared) is passed to the ice sheet model as ablation (i.e., a negative SMB). In ice‐free regions of CISM2, negative SMB from CLM5 is ignored. The SMB definition used in the ice sheet‐land model coupling does not take into account variations of mass within the snow and firn pack. Thus, the SMB received by CISM reflects long‐term changes in ice thickness, but not seasonal‐to‐interannual variations in snow depth. This approximation is valid for equilibrated climate and SMB, but introduces a bias during transient climates, especially in the vicinity of the equilibrium line altitude (ELA). The reason is a delay in the transition from positive or negative SMB, or vice versa, when using SMB that includes snow pack changes, versus using SMB as the difference between ice accumulation and ice ablation.

After annual SMB is accumulated in CLM5 for each glacier‐covered EC, the SMB is remapped from the coarse land model grid to the higher‐resolution ice sheet model grid through trilinear interpolation and renormalization. The remapping procedure targets (a) local accuracy, (b) smooth SMB gradients without any imprint of the coarse land model grid, (c) preservation of the spatial distribution of accumulation and ablation areas, and (d) conservation of mass. To reach all these targets, the downscaling algorithm proceeds as follows:

1. The CESM2 coupler (the interface between the land and ice‐sheet models) accumulates and averages the SMB in each land model grid cell over the coupling interval (annually for synchronous land/ice‐sheet coupling).
2. SMB in each EC is (horizontally) bilinearly interpolated from the land model to the ice‐sheet model grid.
3. In each ice‐sheet grid cell, the SMB is linearly interpolated in the vertical between adjacent ECs to the appropriate surface elevation in the ice‐sheet model.
4. Mass discrepancy (%) following these interpolations is quantified by separately evaluating the total area‐weighted accumulation and ablation on the source grid (i.e., the land model, accounting for elevation classes) and destination grid (i.e., the ice‐sheet model). This results in two normalization factors, one for the accumulation region and one for the ablation region. For the coupled GrIS simulations described here, the mass discrepancy usually is less than 10%, and thus the normalization factors fall between 0.9 and 1.1.
5. In each ice‐sheet grid cell, the SMB is multiplied by the appropriate normalization factor to ensure mass conservation.

### 3.2 Freshwater Fluxes From the Ice Sheet to the Ocean

Surface runoff, ice discharge, and basal melt comprise the freshwater flux from the ice sheet to the ocean. Marine‐terminating glaciers discharge ice directly to the ocean. Thus, the coupling is only from ice to ocean, and no information is passed from the ocean to the ice sheet. As a result, CESM does not yet simulate ocean‐forced melting of marine‐terminating glaciers, as described by Goelzer et al. (2020) and Slater et al. (2020) for standalone GrIS experiments for ISMIP6. Surface runoff consists of liquid water at the ice sheet surface that is not refrozen in the snow pack. Surface runoff fluxes are computed in the surface hydrology module of CLM5. Basal melting occurs where the base of the ice sheet is at the pressure melting point due to frictional and geothermal heat. Basal melt flux is computed in CISM2 (for details, see Lipscomb et al., 2019) and sent to the ocean, where it is treated in the same way as surface runoff from CLM5.

The relevant aspects of water fluxes to the ocean, from a coupling perspective, are the source (i.e., land or ice‐sheet model), timing, and phase (i.e., solid or liquid). Surface runoff fluxes are calculated in the land model and coupled to the ocean on hourly time scales. The ice sheet model, on the other hand, computes annual ice discharge and basal melting rates, which are supplied to the ocean at a constant rate throughout the following year (without seasonal modulation). Solid ice fluxes from ice discharge are melted instantaneously (temperature of ice is assumed to be 0°), and the energy for the phase change is extracted from the global surface ocean. For liquid fluxes, the temperature difference between the liquid‐phase water flux and the ocean temperature is assumed to be negligible, such that no net heat transfer occurs. Since the water volume in POP2 is fixed, the ocean model converts freshwater fluxes to virtual salinity fluxes.

Water flux routing depends on the model component. Surface runoff from the land model is routed downhill by the river transport model (MOSART) to the ocean. Once on the ocean grid, the water is distributed in the upper three model levels (upper 30 m) using an estuary box model (EBM; Sun et al., 2017). Solid ice discharge and basal melt fluxes from the ice‐sheet model are sent to the ocean using nearest‐neighbor conservative regridding. Solid‐phase water fluxes are distributed horizontally in the surface ocean using a Gaussian distribution with maximum radius of 300 km. This horizontal distribution mitigates unrealistic local frazil sea‐ice growth caused by extraction of heat to melt ice, especially in winter when the ocean surface is near the freezing point.

### 3.3 Dynamical Land–Ice Sheet Mask

The advance and retreat of ice sheet margins change land‐surface properties (e.g., surface albedo), which in turn impacts the full range of surface processes. Each CLM5 grid cell can represent several different land units (e.g., vegetated and glaciated surface types) at the same time. Ice‐sheet/climate coupling thus requires accommodation for dynamic (interactive or time‐evolving) land units, as the ice sheet margin changes.

Dynamic land units are implemented as follows. In each CLM5 grid cell, glacier elevation classes are initialized in accordance with the topography in CISM2. As the ice‐sheet topography evolves, the coupler remaps the new ice‐sheet geometry from CISM2 to CLM5. The coupler also uses the ice sheet extent in CISM2 to recompute the fractional glacier coverage (in each elevation class) in each CLM5 grid cell that overlaps the CISM2 domain. Adjustments are made to the total glacier area of the grid cell, as well as the glacier area and mean topographic height in each elevation class. CLM5 land units are updated at the CISM2 coupling frequency (i.e., annually for synchronous climate/ice‐sheet coupling).

The transition from vegetated to glaciated land unit when the ice sheet margin advances (or vice versa, from glaciated to vegetated when the margin retreats) requires conservation of mass and energy in each CLM5 grid cell. As the total glacier area in each CLM5 grid cell changes, the area of each subgrid column (i.e., elevation class) also changes. Each column in CLM5 has a different subsurface water, energy, carbon, and nitrogen content. Specifically, vegetated columns have soil layers, while glacier columns have subsurface ice layers. CLM5 does not adjust the below‐ground states for water and energy when the fractional areas of land units change. Instead, correction fluxes are applied to ensure conservation of mass and energy. Runoff fluxes (either positive or negative) are generated to conserve liquid water and ice, and sensible heat fluxes are generated to conserve energy. These correction fluxes are typically small compared to the physical fluxes of water and energy, and they are distributed evenly throughout the following year to MOSART and CAM6, respectively. Regarding soil carbon and nitrogen, glacier land units typically do not track this. When the ice sheet retreats, it is assumed to contribute zero carbon and nitrogen to the newly vegetated land unit's soil column. When the ice sheet advances, however, the glacier land unit will store the carbon and nitrogen from the previously vegetated land unit. The carbon and nitrogen contents remain unchanged until the area deglaciates again. Further details can be found in Leguy et al. (2018), Section 6.3 and Andre et al. (2020), Section 2.27.

When the ice sheet retreats, the vegetation types that regrow on deglaciated land are determined by the input map of PFTs. The default PFT distribution in CESM2 is based on present‐day observations (Bonan et al., 2002). By default, land areas under the GrIS are prescribed as bare ground. A more realistic approach is to change the vegetation to be consistent with the evolving climate, but this requires running a vegetation model offline (e.g., Kaplan et al., 2003) and manually updating the PFT map.

Running with POP, the land‐sea mask must remain fixed at runtime. The MOM6 ocean model (Adcroft et al., 2019), which will replace POP in future CESM versions, can simulate an evolving ocean‐ice sheet boundary, but is not yet supported for coupled CESM‐CISM applications with realistic ice sheets.

### 3.4 Dynamical Ice Sheet Surface Topography

As ice‐sheet geometry evolves over long timescales, changes in topography can influence atmospheric circulation. For century‐scale or longer coupled ice‐sheet/Earth system simulations, this becomes an important feedback. In the case of CESM2, this means updating the topographic boundary conditions. The topography updating routine modifies CAM6's boundary conditions and restarts files based on ice‐sheet elevation changes in CISM2. The required files for this workflow are a source file of CISM2 topography, a destination file of surface boundary conditions on the CAM6 grid, and a high‐resolution topography data set to inform on elevations over non‐ice‐sheet‐covered land surface regions. In our case, this data set is the GMTED2010, a 30″ (nominally 1 km resolution) global Digital Elevation Model (DEM) from USGS on a rectilinear latitude‐longitude grid (Danielson & Gesch, 2011).

The topography updating procedure is as follows:

1. The regional ice‐sheet topography from the 4‐km rectangular CISM grid is bilinearly interpolated and merged with the full global 30‐s DEM data set.
2. The NCAR topography generation software for unstructured grids (NCAR_topo; Lauritzen et al., 2015) computes the surface topography variables and maps them to the CAM 1° finite volume grid. The mapped variables are surface geopotential (*PHIS*); the standard deviation of large subgrid‐scale topography (*SGH*, approximately >3 km and <grid‐scale); and the standard deviation of small subgrid‐scale topography (*SGH30*; approximately <3 km). The latter two variables estimate subgrid surface roughness, and are used in the CAM6 parameterizations of orographic gravity wave drag and the Beljaars turbulent orographic form drag, respectively.
3. Updated CAM surface topography variables are merged into the standard CAM default restart file and topographic boundary condition in preparation for the next run period.

## 4 Model Demonstration

To demonstrate the coupling across model components in CESM2‐CISM2, we compare the simulated GrIS energy and mass fluxes in a pre‐industrial simulation and a simulation under a high CO_2_ forcing scenario. Given that simulations with this model are computationally demanding, we have used simulations that are also used in a scientific application publication. We have tried to avoid any scientific relevance of the demonstrated analysis, as the aim is to illustrate two contrasting climate states, and show the coupling features that enable the model to simulate a transient but coherent climate between them. The analysis focuses on behavior of the four coupling aspects that are described in Section 3 (SMB, ocean freshwater budget from Greenland, dynamic ice sheet margins, and surface topography updating).

### 4.1 Experimental Set‐Up and Model Configuration

The pre‐industrial (hereafter PI) steady‐state simulation is designed following the CMIP6 guidelines as specified in Eyring et al. (2016), with a fixed atmospheric CO_2_ concentration of 284.7 ppmv (parts per million by volume). The idealized high‐CO_2_ scenario (hereafter hCO_2_) is a 350‐year simulation that starts with a transient component in which the atmospheric CO_2_ concentration increases by 1% per year for 140 years (until the CO_2_ concentration is 1,140 ppmv, or four times the pre‐industrial value), after which it is held fixed until model year 350. The experimental set‐up is described in detail in Nowicki et al. (2016).

Both simulations are initialized from the spun‐up pre‐industrial climate/ice‐sheet state described in Lofverstrom et al. (2020). In this state, the GrIS volume is about 12% larger than the observed present‐day ice sheet (estimated 7.4 m sea‐level equivalent; Morlighem et al., 2017), and the residual drift is ∼0.03 mm SLE/yr of GrIS mass gain. The simulated ice sheet area is 15% larger than observed, with most of the differences in the north. See Lofverstrom et al. (2020) for a more detailed comparison with observations and regional climate model reconstructions.

The model configuration used here is the same as in CESM2 without an evolving GrIS, except for several minor modifications, as permitted by the ISMIP6 guidelines (Nowicki et al., 2016). These modifications were implemented to limit the over‐growth of the GrIS during the multi‐millennial spin‐up (Lofverstrom et al., 2020). First, the CLM5 default precipitation repartitioning as described in Section 3.1 is modified so that rain falling in sub‐freezing conditions immediately runs off to the ocean rather than being converted to snow. This change compensates for excessive precipitation in the model (van Kampenhout et al., 2020). Second, we reduced the magnitude of the elevation feedback by turning off the downscaling of longwave radiation in the EC scheme. Also, we limited excessive ice sheet expansion by specifying that, for non‐glaciated areas, ice sheet inception is only allowed in grid cells directly adjacent to the main ice sheet. The calving parameterization is based on a flotation criterion: any floating ice is assumed to calve immediately. Based on the evolving ice sheet surface topography, the topography is updated annually at runtime in the land model, and is updated offline in the atmosphere model every 10 years.

### 4.2 Results

In the following analysis, the mean pre‐industrial state is compared to a 20‐year segment of the idealized high‐forcing simulation (years 201–220). In this segment, the climate has warmed significantly and ice sheet changes are noticeable. The global mean surface temperature is 7 K warmer than pre‐industrial. The GrIS area and volume are 7% and 3.8% smaller than pre‐industrial, respectively (Table 1). The substantial mass loss between the PI and hCO_2_ simulations (−13 Gt yr^−1^ compared to −1,506 Gt yr^−1^; Table 1) reflects rapid GrIS deglaciation in the hCO_2_ case. Conversely, ice discharge decreases in the hCO_2_ case, as the margin thins and migrates inland from the coast (Muntjewerf, Sellevold, et al., 2020).

**Table 1:** Climate and Ice Sheet Variables: Mean [Standard Deviation] Over the Selected 20‐Year Period

	Pre‐industrial	High forcing
Global		
Mean 2‐m temperature (K)	287	294
GrIS		
Ice area (m^2^)	1.97 × 10^12^	1.82 × 10^12^
Ice volume (m^3^)	3.23 × 10^15^	3.11 × 10^15^
Surface mass balance (Gt yr^−1^)	585 [85]	−1,293 [217]
Ice discharge (Gt yr^−1^)	574 [5]	196 [19]
Basal mass balance (Gt yr^−1^)	−24 [0]	−17 [0]
Total mass balance (Gt yr^−1^)	−13 [84]	−1,506 [214]
Rate of SLR (mm yr^−1^)	0.03 [0.23]	4.18 [0.59]
Greenland		
Runoff DJF (Gt yr^−1^)	95 [22]	185 [31]
Runoff JJA (Gt yr^−1^)	857 [118]	6,197 [533]

#### 4.2.1 Surface Mass Balance

**Figure 3:**
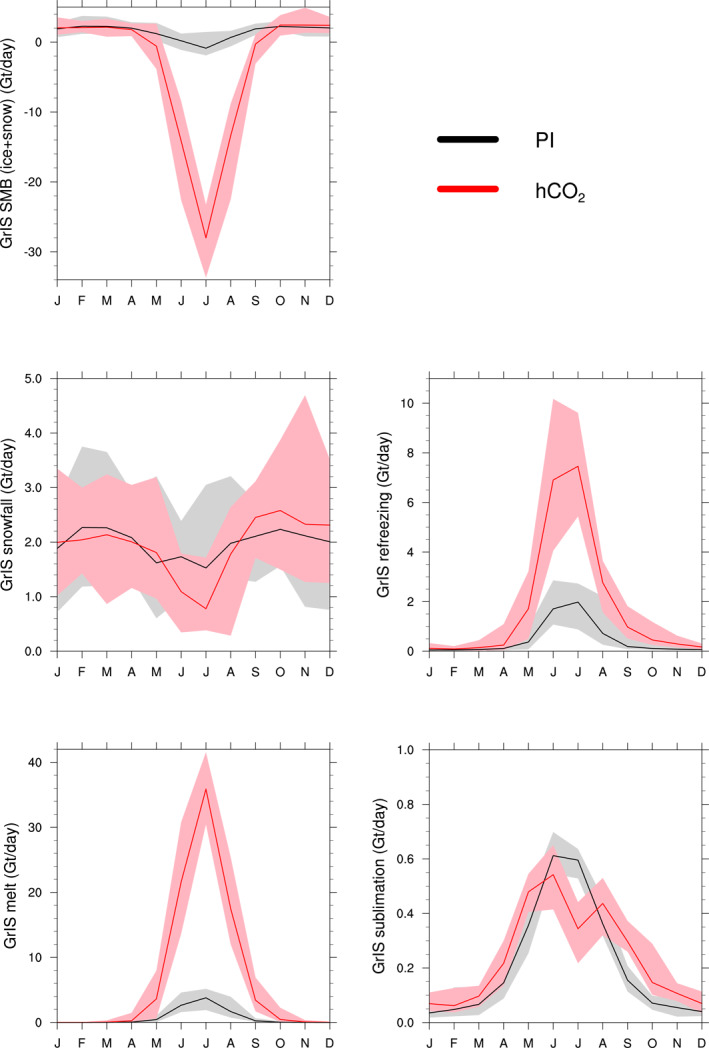
Climatology of the GrIS‐integrated surface mass balance (Gt day^−1^) and components (SMB = snowfall + refreezing − melt − sublimation) from the land model CLM5 for (black) pre‐industrial forcing and (red) high atmospheric CO_2_ forcing. Solid line is the mean, and shaded area is the range over the 20‐year period. CLM5, Community Land Model Version 5; GrIS, Greenland Ice Sheet; SMB, surface mass balance.

Figure 3 shows mean annual cycles of GrIS‐integrated SMB components. The PI SMB is positive for 10 months of the year. The most negative SMB is in July (*μ* [*σ*]: −1.16 [0.95] Gt day^−1^), and the summer mean snowfall of 1.73 [0.55] Gt day^−1^ is slightly less than the annual value of 1.97 [0.27] Gt day^−1^). Refreezing of meltwater within the snow and firn layers contributes substantially to net SMB (2.71 [0.88] Gt day^−1^). However, the sum of melt and sublimation (4.98 [1.35] Gt day^−1^ and 0.61 [0.03] Gt day^−1^, respectively) is larger than the sum of snowfall and refreezing, resulting in the slightly negative net SMB.

In the hCO_2_ simulation, the number of months with a positive SMB decreases to seven, and the annual mean SMB is negative (Table 1). The extended melt season is characterized by both an earlier onset of the melt season (April vs. May in the PI) as well as a later ending (October–November vs. September in the PI). As in the PI run, the largest negative SMB is simulated in July (−27.09 [2.82] Gt day^−1^), with melt as the main contributor (35.78 [2.89] Gt day^−1^). Not all additional melt water contributes to the surface mass loss, as the amount that refreezes increases as well (to 7.44 [0.98] Gt day^−1^). Snowfall decreases in the summer months, and increases in the fall and early winter. Sublimation decreases in the summer and increases during the rest of the year.

The range of melting in these simulations agrees with a recent study by Tedesco et al. (2013), which put the extreme melt year of 2012 in historical perspective. Mean daily melt of about 6 Gt day^−1^ in July was modeled by MAR for the period 1958–2011, with a maximum of about 16 Gt day^−1^. The modeled extreme melt in 2012 was 22 Gt day^−1^, with is less negative than the CESM2.1 July average in hCO_2_, but far more negative than PI values.

**Figure 4:**
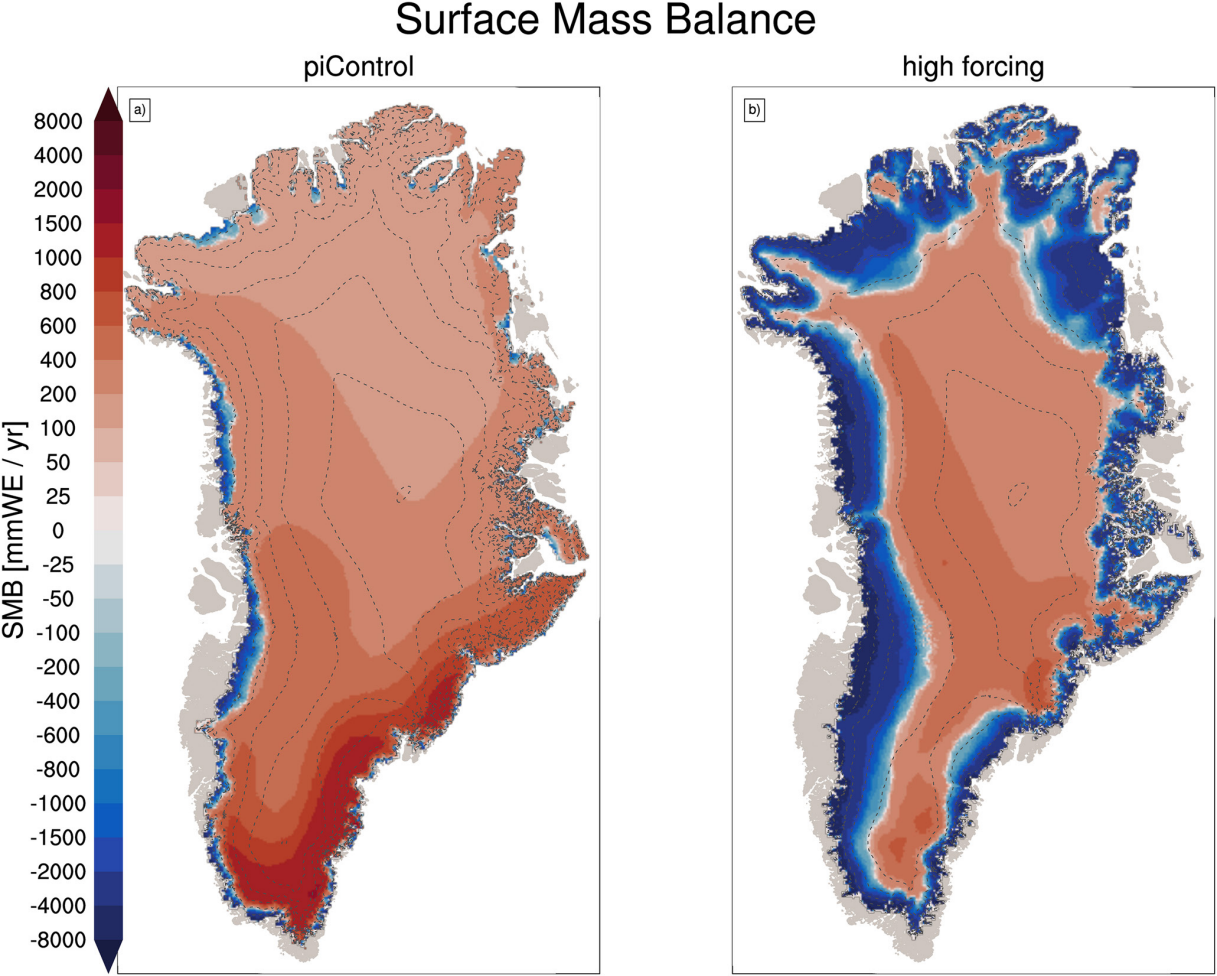
20‐year mean surface mass balance (mm WE yr^−1^) from the ice sheet model CISM2 for (a) pre‐industrial forcing and (b) high atmospheric CO_2_ forcing. Accumulation zone in red, ablation zone in blue. Dashed lines show ice sheet elevation contours at 500‐m intervals. CISM2, Community Ice Sheet Model version 2.

Figure 4 maps the climatological SMB in CISM2.1 for the two simulations. For PI, the model simulates ablation zones around all margins, with the widest ablation zone in the southwest. High SMB along the southeast coast is well represented, due to high precipitation from simulated storm tracks impinging on Greenland from the North Atlantic. The interior and the northern half of the ice sheet are much drier than the south, in accordance with present‐day measurements and reconstructions (Ettema et al., 2010; Noël et al., 2018).

For the PI run, the GrIS‐integrated annual‐mean SMB is 585 [85] Gt yr^−1^ (Table 1). This is within the bounds of the SMB at the end of the spin‐up simulation (591 [83] Gt yr^−1^; Lofverstrom et al., 2020). van Kampenhout et al. (2020) used observations and regional climate models to evaluate the GrIS climate and SMB for the period 1961–1990 in CESM2.1‐only runs with non‐evolving, present‐day observed topography. The multi‐ensemble mean GrIS SMB from this study is 508 [73] Gt yr^−1^, slightly lower than for the PI CESM2.1‐CISM2.1 run and in good agreement with the reconstruction by the regional climate model RACMO forced with reanalysis.

In the hCO_2_ run, the integrated SMB decreases to −1,293 Gt yr^−1^ (Table 1). The ablation areas expand, and the average ELA rises above 2,000 m. The SMB slightly increases in the high interior regions because of enhanced snowfall (Muntjewerf, Sellevold, et al., 2020; Sellevold & Vizcaino, 2020).

**Figure 5:**
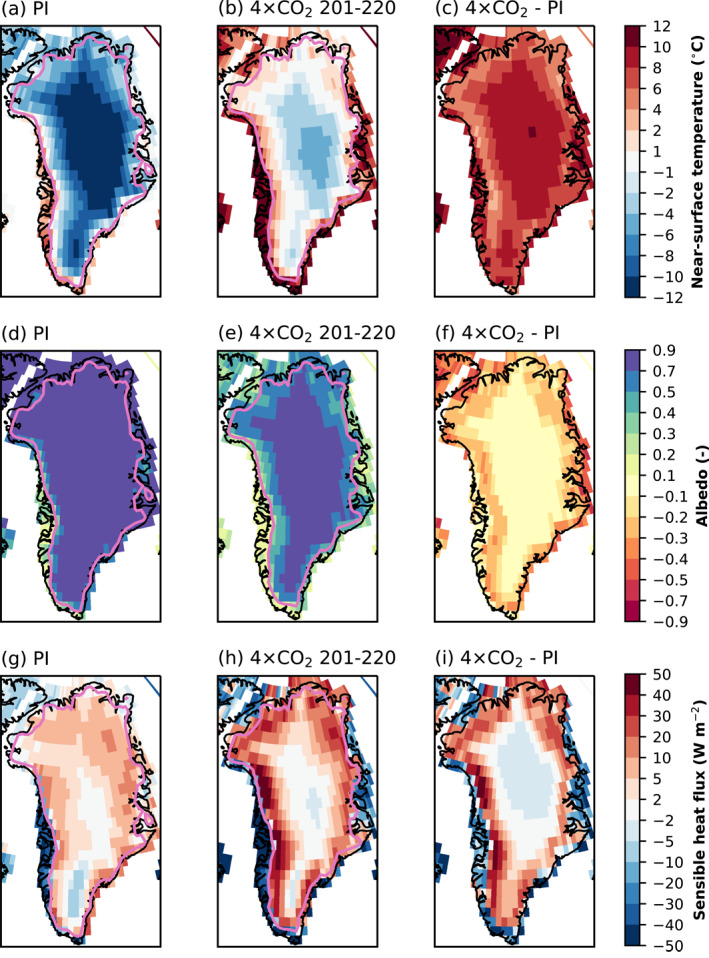
Simulated summer (JJA) Greenland climate from the land model CLM5 for pre‐industrial forcing (first column), high atmospheric CO_2_ forcing (hCO_2_, years 201–220, second column), and difference (third column): (a–c) near‐surface temperature (°C), (d–f) albedo, (g–i) sensible heat flux (positive from atmosphere to surface, W m^−2^). CLM5, Community Land Model Version 5.

Figure 5 shows how the SMB simulation is coupled with the simulated climate over Greenland. In the PI, the average summer (JJA) near‐surface temperature is at least several degrees below the melting point for most of the GrIS except in the ablation areas, where it is at or near the melting point (Figure 5a). In hCO_2_, temperatures at or above the melting point largely correspond with the ablation areas (Figure 5b). The map of temperature increase is not uniform (Figure 5c), as the ablation areas (where the surface temperature is held at the melting point) warm less than the interior. Over the ablation areas (Figure 4), albedo decreases in hCO_2_ from greater exposure of bare ice (Figures 5d–5f). The turbulent heat flux from the atmosphere to the ablation areas increases (Figures 5g–5i) as the atmosphere warms well above the melting point (Figures 5b and 5c), while the surface is constrained to the melting temperature.

#### 4.2.2 Freshwater Fluxes

**Figure 6:**
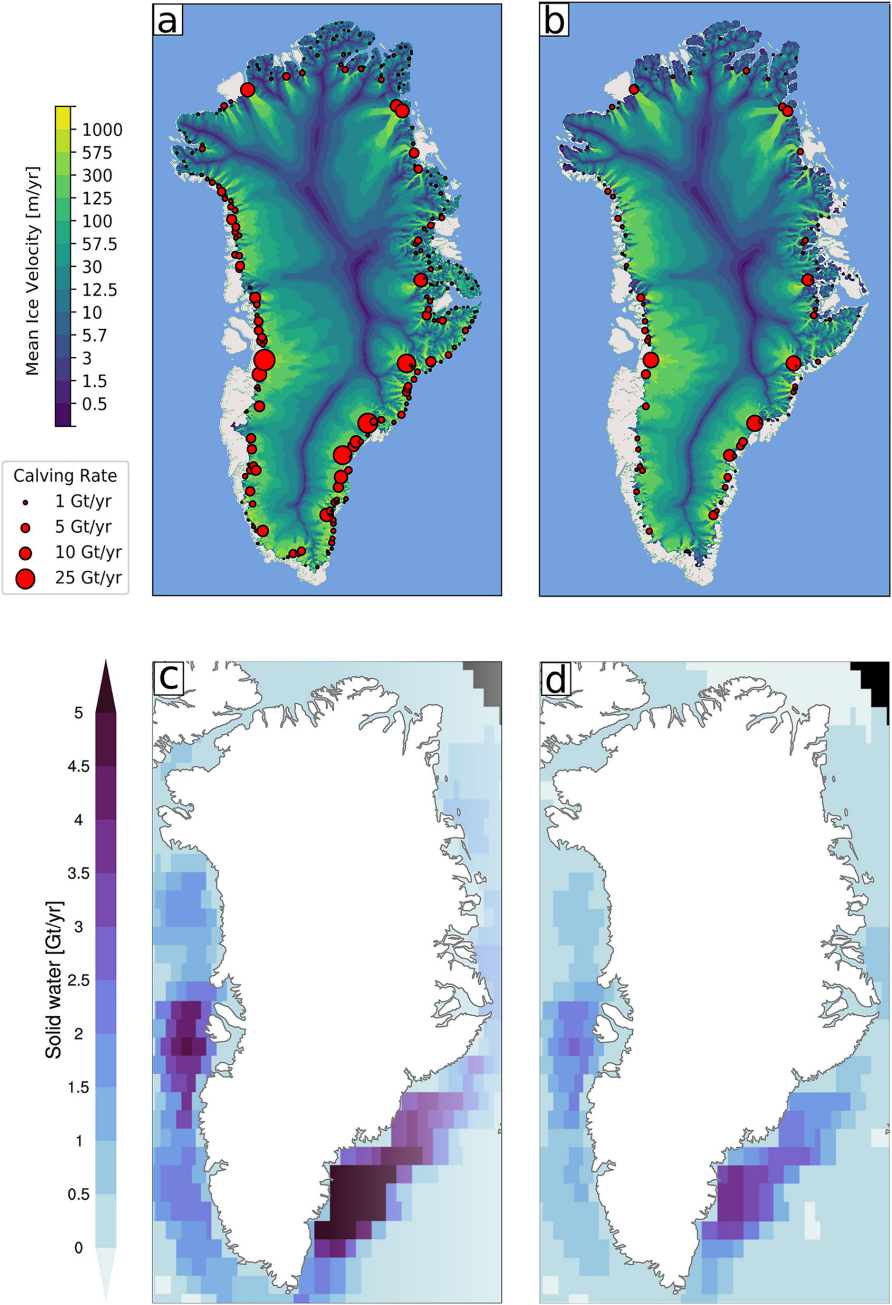
Simulated solid freshwater fluxes from Greenland (ice discharge) to the ocean. Top: CISM2 dynamic ice discharge for the modeled outlet glaciers (red circles; Gt yr^−1^) and surface ice velocity (background, m yr^−1^) for (a) pre‐industrial forcing and (b) high atmospheric CO_2_ forcing. Mean over the 20‐year period. Bottom: POP2 solid phase freshwater input (Gt yr^−1^) for (c) pre‐industrial forcing and (d) high atmospheric CO_2_ forcing. Mean over the 20‐year period. CISM2, Community Ice Sheet Model version 2.

The simulated fluxes to the CESM ocean component include ice discharge in the solid phase, along with surface runoff and basal melt in the liquid phase. Basal melt is not discussed here, given that it is an order of magnitude smaller than ice discharge and surface runoff, and the simulation does not include ice shelves or subshelf melt. Figure 6a shows CISM ice discharge in the PI. The largest contributions are from Jakobshavn in the west, and from Helheim and Kangerlussuaq in the southeast. Muntjewerf, Petrini, et al. (2020), Supporting Information compared the simulated ice discharge in a CESM2.1‐CISM2.1 historical simulation with observations for the recent decades. In the hCO_2_ segment (Figure 6b), the ice sheet margin has retreated inland, and ice has thinned around the margins. As a result, the ice discharge decreases by a factor of three (Table 1). Ice velocities increase in the intermediate area between the margin and the high interior as a result of increased topographic gradient due to greater thinning in the margins. The ocean receives the solid water flux corresponding to the ice discharge (Figures 6c and 6d), with the highest inputs in the southeast and northeast.

**Figure 7:**
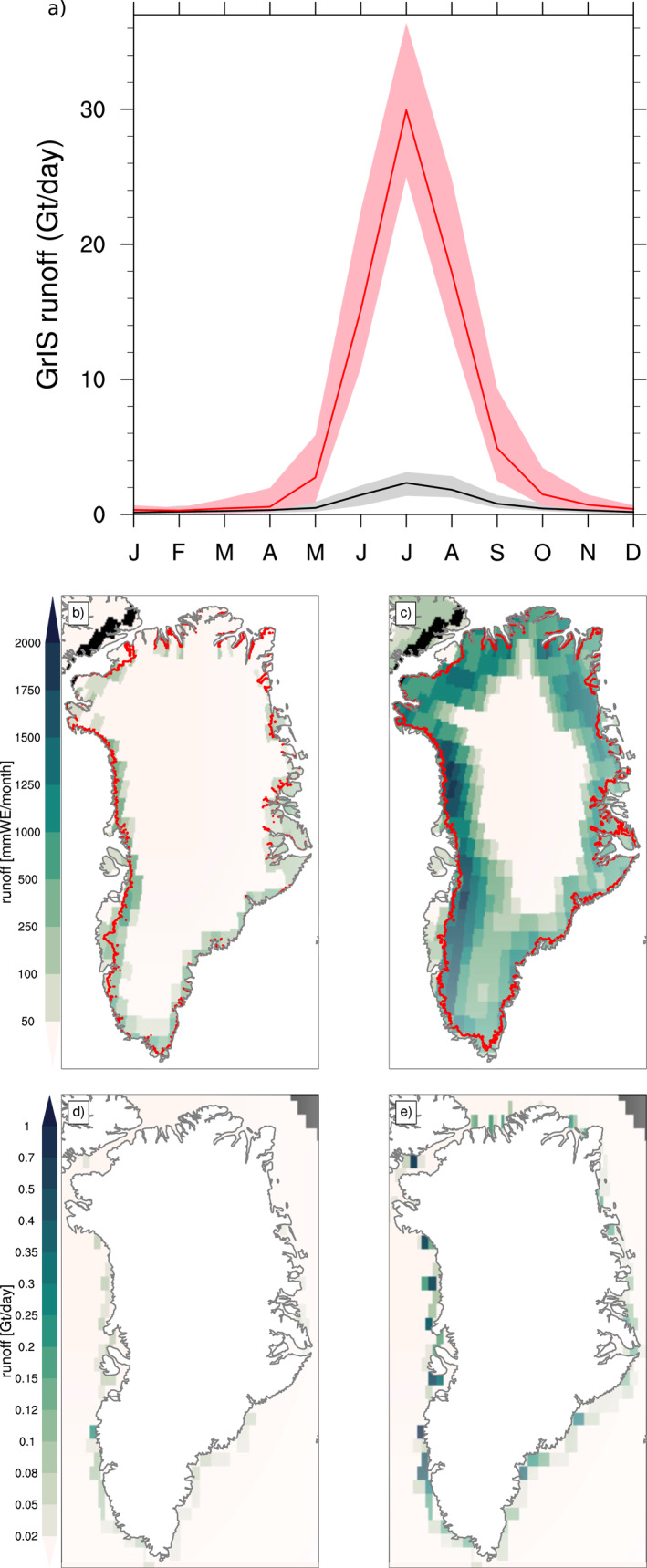
Runoff: (a) climatology in CLM5 of GrIS integrated runoff (Gt day^−1^) for (black) pre‐industrial forcing and (red) high atmospheric CO_2_ forcing. Solid line is the mean, and shaded area is the range over the 20‐year period. Spatial map of JJA Greenland runoff as generated in the land model CLM5 (mmWE month^−1^) for (b) pre‐industrial forcing and (c) high atmospheric CO_2_ forcing; mean over the 20‐year period. Red line denotes the ice sheet margin. Spatial map of JJA runoff from Greenland as received by the ocean model POP2 (Gt day^−1^) for (d) pre‐industrial forcing and (d) high atmospheric CO_2_ forcing; mean over the 20‐year period. CLM5, Community Land Model Version 5; GrIS, Greenland Ice Sheet.

The PI annual average runoff is 351 [45] Gt year^−1^. On average, there is five times more runoff in the hCO_2_ run than in the PI run (Table 1). This is due to a much higher peak in summer melting, as seen in the seasonal cycle of GrIS‐integrated runoff in Figure 7a. Figure 7 shows the spatial extent of July runoff for the PI (left panel) and hCO_2_ (right panel). The spatial pattern of runoff corresponds closely with the ablation simulation in Figure 4, with ablation zones being relatively narrow in the PI but extending far inland in the hCO_2_. Figures 7d and 7e show where the JJA runoff enters the ocean in the PI and hCO_2_, respectively. Consistent with the largest increases in ablation area and magnitude, the largest runoff increases in the hCO_2_ are found along the western and southeastern coasts.

#### 4.2.3 Margin Advance and Retreat

**Figure 8:**
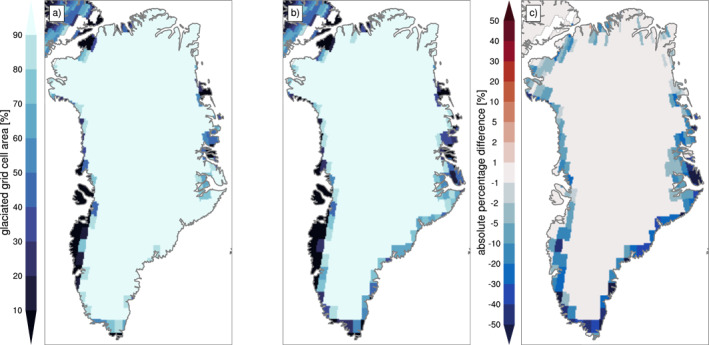
Simulated percentage of glaciated fraction (sum of all ECs) per grid cell in the land model CLM5 for (a) pre‐industrial forcing, (b) high atmospheric CO_2_ forcing, and (c) the difference of (a) and (b). CLM5, Community Land Model Version 5.

The difference in GrIS area between hCO_2_ and PI is −0.15 × 10^6^ km^2^ (−7.6%) (Table 1). The loss of CISM ice‐covered area in the hCO_2_ case redistributes EC areas within CLM grid cells and replaces glacier land units with vegetation. Figure 8 compares the percentage of grid‐cell glaciated fraction in the PI and hCO_2_ simulations. The largest changes are found along the southern and eastern margins. This strongly affects land surface properties (e.g., albedo) and the energy exchange with the atmosphere in the deglaciated areas (Figure 5). The largest albedo changes over Greenland correspond to deglaciated areas (compare Figure 8 and Figures 5d–5f). The retreat of the ice sheet (e.g., in mid‐eastern Greenland) reverses the sign of the summer sensible heat flux from positive (surface to atmosphere) to negative (atmosphere to surface) (Figures 5g and 5h), since incoming radiation can warm the tundra above the melting point (Figure 5b), while glaciated surfaces are held at the melting point (Figure 5a). Further changes in the Greenland climate related to ice sheet margin retreat for the extension of the hCO_2_ simulation to year 350 are shown in Muntjewerf, Sellevold, et al. (2020).

#### 4.2.4 Ice Sheet Topography Changes

**Figure 9:**
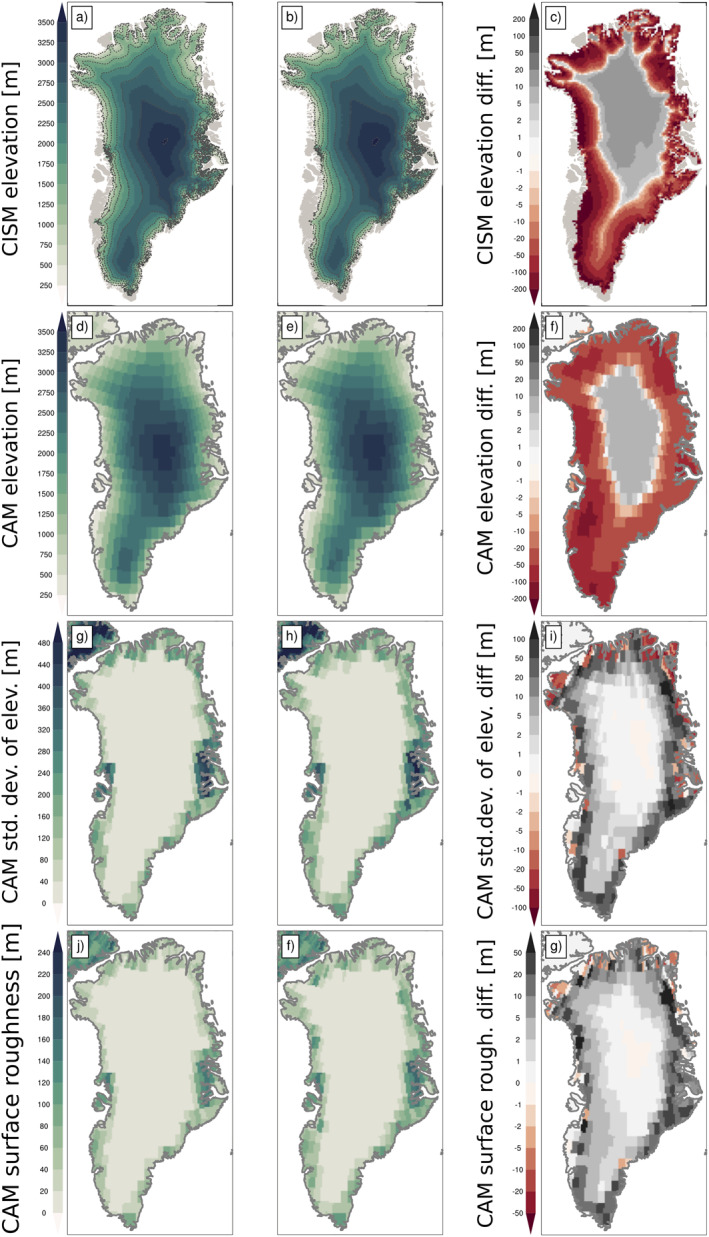
Greenland topography in the ice sheet model CISM2. Surface elevation (m) for (a) pre‐industrial forcing, (b) high atmospheric CO_2_ forcing, and (c) the difference of (a) and (b). Dashed lines are ice sheet elevation contours at 500‐m intervals. Panels (d) and (e) show the surface elevation in the atmosphere model for these two periods, with the difference in (f). Panels (g) and (h) show large subgrid‐scale standard deviation of surface elevation *SGH* (m) in the atmosphere model, with the difference in (i). Panels (j) and (k) show the small subgrid‐scale standard deviation in elevation *SGH30* (m) in the atmosphere model, with the difference in (l). CISM2, Community Ice Sheet Model version 2.

The total GrIS volume decreases by 0.12 × 10^6^ km^3^ (3.7%) (Table 1) in the hCO_2_ run. Figures 9a and 9b show the surface elevation in CISM2, with the greatest thinning along the margins and in the south (Figure 9c). Differences in elevation correspond with the spatial pattern of SMB changes in Figure 4. Figures 9d and 9e show the corresponding surface elevation in the atmosphere model. While the atmosphere model cannot directly resolve small‐scale ice sheet topography changes, these changes are captured in subgrid‐scale topography variance fields: large‐scale (3 km < *σ* < 100 km; Figures 9g and 9h) and small‐scale (*σ* < 3 km) surface elevation variance (Figures 9j and 9k). As ice thins near the margin, the topography gradient increases over the ablation areas, resulting in increases in (both large and small) subgrid elevation variations. Anomalies for the small subgrid‐scale coincide largely in sign with those of large scale, albeit with reduced magnitude, with some exceptions around the areas of ice sheet retreat, particularly in the east. These might be related to the complex sub‐grid topography of the region. As the margin thins and retreats, the elevation differences between high, formerly ice‐covered regions and lower, ice‐free regions decrease. This results in some increases in the fine‐scale topographic gradient.

## 5 Discussion

This study presents the coupled ice sheet and Earth system model CESM2‐CISM2, which includes a higher‐order ice sheet model, an advanced SMB calculation with explicit simulation of albedo and refreezing, and a high‐complexity (“IPCC‐class”) ESM that accounts for simulated changes in ice sheet area, elevation, and freshwater fluxes to the ocean. The coupled model conserves mass and energy, an important feature for robust long‐term simulations of climate and ice sheet changes (Fischer et al., 2014).

The elevation‐class method used for SMB downscaling (Lipscomb et al., 2013; Sellevold et al., 2019), paired with conservative trilinear remapping, is used to bridge the spatial resolution gap between the land and the ice sheet components. Compared with previous model versions (Vizcaino et al., 2013, 2014), the SMB calculation has been substantially improved in CESM2 (Danabasoglu et al., 2020); for example, through the implementation of a wind‐dependent snow albedo scheme, an increased maximum allowed snow depth, and explicit simulation of snow and firn compaction (Van Kampenhout et al., 2017). Advances in the representation of clouds and turbulent fluxes have also contributed to a more realistic Greenland climate and surface melt. CESM2 simulates a realistic GrIS SMB for the historical period and a fixed present‐day ice sheet topography (van Kampenhout et al., 2020) (Noël et al., 2019). The calculation of SMB in the land component permits direct coupling between SMB and climate, as for instance surface albedo affects the atmosphere–surface fluxes (see Figures 4 and 5, where expanded ablation areas result in lower summer surface albedo). This is an improvement over previous modeling efforts in which the SMB was typically calculated offline with input from a climate model (e.g., Aschwanden et al., 2019; Gregory & Huybrechts, 2006; Ridley et al., 2005; Vizcaino et al., 2015).

Compared to standard CESM2 with prescribed ice sheet geometry, the coupled model provides more physically based runoff and ice discharge fluxes to the ocean, as these correspond to the SMB calculation in the land model and the calving flux in the ice sheet model. This enables spatio‐temporally varying ice sheet freshwater fluxes to the ocean, which is important for realistically simulating the ocean response to ice sheet meltwater (Lenaerts et al., 2015). The coupled model features a dynamic (or “time‐evolving”) glacier mask that enables a more realistic representation of land processes in regions that glaciate when the ice‐sheet margin expands (or vice versa, become ice‐free when the margin retreats). These transitions substantially alter the turbulent and radiative surface fluxes (primarily in summer; Section 4.2.1) as shown in the contrast between contiguous glaciated and non‐glaciated areas in Greenland climate reconstructions (Ettema et al., 2010; Van den Broeke et al., 2016). With the current calving parameterization that calves all floating ice, marine‐terminating glaciers can not be simulated. This is a limitation as there are modeling studies that indicate a competition between enhanced ice discharge from glacier acceleration and decreased ice discharge from thinning due to surface mass loss (Goelzer et al., 2020; Slater et al., 2020).

The coupling between ice sheets and the broader Earth system will be further developed in later model versions. Future model developments will include bi‐directional coupling to the ocean, as ocean thermal forcing can drive the retreat of marine‐based ice (Bondzio et al., 2018; Joughin et al., 2012; Khazendar et al., 2019). Other planned improvements include the implementation of a dynamic ice sheet‐ocean mask (Straneo et al., 2013), especially for paleo studies (Meccia & Mikolajewicz, 2018), as well as a dynamic land‐ocean mask that adjusts to changing ice shelf extent over the ocean. These developments will become feasible with the transition from POP to MOM6 (Adcroft et al., 2019) as the CESM ocean component, since MOM6 allows ocean circulation in evolving ice‐shelf cavities.

Also, runoff in the land surface hydrology model could be routed along dynamic ice‐sheet surface gradients, instead of being prescribed based on present‐day topography. Another desirable feature is dynamic vegetation, which can modify the climate through albedo and ecosystem feedbacks (Rombouts & Ghil, 2015; Sturm et al., 2005; Thompson & Koenig, 2018). This feedback is potentially important for long transient climate simulations (>500 years) where both ice sheets and vegetation may change substantially due to low‐frequency changes in external forcing, as in the study by Ziemen et al. (2014).

Finally, we reflect on computational expense. As for all highly complex ESMs with medium to high‐resolution atmosphere and ocean components, the computational cost of CESM2 is high, although the additional cost of running CISM is small (less than 1% of the total model cost). The simulations presented here were run on the Cheyenne high‐performance computer, using 2,160 processors with a load‐balanced processor layout. The model cost is around 3,500 processor‐hours for one simulated year, with throughput of about 17.5 simulated years per wall‐clock day; see Table 1 in Lofverstrom et al. (2020). Since much interest of coupled Earth system/ice sheet simulations lies in centennial or millennial timescales, techniques to reduce costs for long simulations are needed. One possible approach is the development of robust, flexible, modular coupling schemes, using asynchronous or “periodic synchronous” coupling (Mikolajewicz et al., 2007; Pollard et al., 1990; Ridley et al., 2005; Ziemen et al., 2014). A similar approach was adopted to generate the initial conditions for the simulations in this study (Lofverstrom et al., 2020).

## 6 Conclusion

This study describes and demonstrates the interactive coupling of CESM2 with CISM2. The current coupling is bi‐directional throughout the ice sheet‐snow‐climate domain, and uni‐directional through the ice sheet‐ocean interface. This paper covers four primary aspects of the coupling:

- Energy‐balance and SMB calculations in the land model, with SMB remapped to the higher‐resolution ice sheet grid.
- Solid ice discharge from the ice sheet model to the ocean model.
- Dynamic surface types in the land model as the ice sheet advances or retreats.
- Evolution of the land‐atmosphere boundary with changing ice‐sheet surface topography.

We demonstrated the capabilities of the new model by comparing a preindustrial simulation with a transient, high‐CO_2_ simulation. In the warmer climate, the GrIS‐integrated SMB becomes negative for the annual mean, has a much larger amplitude in the seasonal cycle, and expanded ablation zones. With greater melt in low‐elevation regions, the ice sheet margins thin. This results in steeper topographic gradients near the ELA, enhancing ice flow from the interior to the margins (Figures 6a and 6b). The magnitudes of runoff and its seasonal variation increase. At the same time, the ocean receives less ice discharge as marine‐terminating glaciers thin and recede inland. This illustrates the coupling between ice‐sheet melt, ice discharge, and freshwater forcing to the ocean. As the ice sheet retreats, previously glaciated areas become bare soil or vegetated, reducing the surface albedo and modifying the magnitude and/or sign of turbulent heat exchange with the atmosphere. The evolving ice sheet topography alters the mean topography and sub‐grid variability seen by the atmosphere model, modifying the atmospheric circulation.

CESM2‐CISM2 is one of a small number of coupled ice sheet‐climate models participating in the CMIP6‐endorsed ISMIP6 experiments (Nowicki et al., 2016), with studies of 1,850–2,100 historical and SSP5‐8.5 simulations (Muntjewerf, Petrini, et al., 2020), and of preindustrial and transient 1% CO_2_ simulations (Muntjewerf, Sellevold, et al., 2020). These simulations are an important contribution to the international effort to include ice sheets as interactive components of Earth System Models.

## Data Availability

Computing and data storage resources, including the Cheyenne supercomputer (https://doi.org/10.5065/D6RX99HX), were provided by the Computational and Information Systems Laboratory (CISL) at NCAR. CESM2 is an open‐source model, available at: http://www.cesm.ucar.edu/, with out‐of‐the‐box support for coupled modeling over the Greenland domain. The World Climate Research Program (WGCM) Infrastructure Panel is the official CMIP document home: https://www.wcrp-climate.org/wgcm-cmip. The CMIP6 and ISMIP6 simulations are freely available, and accessible via the Earth System Grid Federation (ESGF) data portals.
